# Huntington's Disease in a Patient Misdiagnosed as Conversion Disorder

**DOI:** 10.1155/2018/3915657

**Published:** 2018-02-18

**Authors:** João Machado Nogueira, Ana Margarida Franco, Susana Mendes, Anabela Valadas, Cristina Semedo, Gustavo Jesus

**Affiliations:** ^1^Department of Psychiatry and Mental Health, Setúbal Hospital Center, Rua Camilo Castelo Branco, 2910-446 Setúbal, Portugal; ^2^Department of Neurology, Setúbal Hospital Center, Rua Camilo Castelo Branco, 2910-446 Setúbal, Portugal; ^3^University Psychiatric Clinic, Faculdade de Medicina, Universidade de Lisboa, Avenida Professor Egas Moniz, 1649-028 Lisboa, Portugal

## Abstract

Huntington's disease (HD) is an inherited, progressive, and neurodegenerative neuropsychiatric disorder caused by the expansion of cytosine-adenine-guanine (CAG) trinucleotide in Interested Transcript (IT) 15 gene on chromosome 4. This pathology typically presents in individuals aged between 30 and 50 years and the age of onset is inversely correlated with the length of the CAG repeat expansion. It is characterized by chorea, cognitive deficits, and psychiatric symptoms. Usually the psychiatric disorders precede motor and cognitive impairment, Major Depressive Disorder and anxiety disorders being the most common presentations. We present a clinical case of a 65-year-old woman admitted to our Psychiatric Acute Unit. During the 6 years preceding the admission, the patient had clinical assessments made several times by different specialties that focused only on isolated symptoms, disregarding the syndrome as a whole. In the course of her last admission, the patient was referred to our Neuropsychiatric Team, which made the provisional diagnosis of late-onset Huntington's disease, later confirmed by genetic testing. This clinical vignette highlights the importance of a multidisciplinary approach to atypical clinical presentations and raises awareness for the relevance of investigating carefully motor symptoms in psychiatric patients.

## 1. Introduction

Huntington's disease (HD) is an inherited, progressive, and neurodegenerative neuropsychiatric disorder characterized by abnormal movements, cognitive deficits, and neuropsychiatric impairment with a worldwide service-based prevalence of 2.71 per 100,000 (95% CI: 1.55–4.72), based on a meta-analysis [1].

HD is caused by the expansion of cytosine-adenine-guanine (CAG) trinucleotide at the coding region of the IT 15 gene on chromosome 4. Patients with more than 36 repeats will develop the disease, and the age of onset is inversely correlated with the length of the CAG repeat expansion [2].

Individuals who are diagnosed with HD are typically aged between 30 and 50 years and have an average life expectancy of 15–30 years after diagnosis.

Regarding the clinical presentation, chorea is the major neurologic finding, consisting in brief, abrupt, irregular, and unpredictable involuntary movements, which potentially leads to disturbances in posture, gait, and balance, resulting in increased risk of falls. There is also cognitive decline, usually beginning as attentional deficits, followed by onset of dementia [3]. Psychiatric symptoms found in HD include isolated symptoms such as apathy, irritability, or incipient memory loss as well as full blown syndromes, such as Major Depressive Disorder or anxiety disorders in 33–76% of patients. Psychotic disorders and mania-like symptoms are rarer, affecting 3–11% [4].

The onset of serious motor and cognitive symptoms is often late in HD and psychiatric symptomatology often precedes motor symptoms by many years. Longitudinal research in the PREDICT-HD [5] and other clinical programs [6] indicates that HD patients develop subtle changes decades before classical presentation. These include cognitive, functional, and psychiatric symptoms, and altered brain morphology and connectivity and even subtle motor deficits [7]. Cross-sectional studies [8, 9] reported that sadness and depressed mood are early symptoms of HD that peak during the early motor symptomatic phase whereas significantly lower rates of depression are present in advanced stages of the disease.

A large international study [10] confirmed that depression, irritability/aggression, obsessive compulsive behaviors (OCBs) and apathy are highly prevalent neuropsychiatric symptoms in HD population. By contrast, the prevalence of psychosis in the same sample was low.

Moreover, the presence of neuropsychiatric symptoms was associated with a positive psychiatric history; particularly a past episode of depression was associated with manifestation of neuropsychiatric symptoms in HD.

However, there is still a dearth of data regarding psychiatric manifestations and care in HD.

## 2. Material and Methods

Literature revision was made using PubMed database under the following specific terms: “Huntington, Depressive symptoms, Prodromal symptoms, Mood, Gait, and Treatment”, as well as studies of epidemiology, etiology, and clinical presentation.

## 3. Case Report

We present the case of a 65-year-old Caucasian woman with no previous history of psychiatric disease until 2012 (60 years of age). At this point, she complained to her family doctor about low mood, reduced energy, and anhedonia. She was prescribed Sertraline 50 mg once a day and maintained the treatment for about 3 years with partial improvement, mainly in the first year.

At 63 years of age, she started complaining to her family doctor about nonspecific limb weakness without movement difficulties. A few months later (April 2015), she was admitted to hospital with a myocardial infarction and was discharged 5 days later with full recovery of coronary perfusion. She was followed in cardiology outpatient clinic for about one year and was discharged because of progressive noncompliance with medical treatment and prescribed medical exams.

During the months that followed the myocardial infarction, her depressive and motor symptoms gradually worsened, leading to eleven visits to the Psychiatric Emergency Room Service (ERS), where she was assessed by Psychiatry and Neurology with complaints of anxiety, low mood, social isolation, deficits in memory retrieval, unspecific pain, and limb weakness with progressive development of abnormal gait. She left the ERS before full medical evaluation multiple times and never accurately followed medication changes that were suggested.

After several visits to the ERS, she was referred to Psychiatry and Neurology outpatient clinics. At Psychiatry consultation, she was, once again, medicated for anxiety and depressive mood, with Duloxetine 30 mg and Gabapentin 300 mg daily. One year later, her condition worsened, which motivated addition of Mirtazapine 30 mg and titration of Gabapentin up to 600 mg, again without proper response.

Simultaneously, she was evaluated in a Neurology appointment because of diminished force and limitation of limb movements. The neurological examination noted unspecific wide-based gait, enhanced bilateral reflexes, and abolished bilateral postural sensitivity. Blood samples showed low folic acid (3,9 ng/mL), which was considered as partial justification for neurological findings and reposition with folic acid was initiated. Additionally, more exams were requested.

One month before the admission at our Acute Inpatient Unit (AIU) that leads to the diagnosis of HD, the patient was, once again, evaluated in the Psychiatric ESR. On observation, the patient showed worsening of mood status, increased pressure of speech, pseudo hallucinations (patient described the following: “a TV host speaks with me, he guides me and tells me to do certain things”), and suicidal ideation. She was started on Valproic Acid 1000 mg and Quetiapine 400 mg and Mirtazapine was suspended. However, behavior changes were maintained and even aggravated (she took her clothes off at the window, broke some lamps at home and was aggressive to her sister), eventually leading to the admission to the psychiatric ward.

The mental state examination at our unit showed low mood and partial disorientation in time. She was unable to provide dates of important life events saying repeatedly “I don't remember” and scored 14/30 at Mini-Mental State Examination (MMSE). Because of her depressive symptoms and cognitive changes, diagnosis of Major Depressive Disorder/Pseudodementia was assumed and she was medicated with Mirtazapine 30 mg and Quetiapine 200 mg. She was discharged 12 (twelve) days later with improved mood, but she still presented abnormal sustained gait, which was interpreted as a comorbid Conversion Disorder.

The Computed Tomography (CT) and Electromyography did not reveal any significant alterations. Therefore, at the subsequent Neurology appointment, patient was discharged with diagnosis of Conversion Disorder.

After hospitalization, she did not follow the prescribed medication and her abnormal behavior was maintained, such as not cleaning her house and throwing clothes away, so she was referred again to the Psychiatric ERS. At the mental state examination she endorsed soliloquies, depressive mood with catathymic delusions, and suicidal ideation and was disoriented to time and place. For that reason, she was readmitted to the Psychiatry ward on March 2017.

During hospitalization the abnormal gait and movements were still obvious; therefore the case was referred to the Neuropsychiatry Team (NT). The NT considered that the psychiatric symptoms of the patient, in addition to the hyperkinetic motor ones, especially in the upper limbs and cervical area (chorea), were in favor of the diagnosis of HD. Genetic test revealed 39 (±2) triplets on one huntingtin allele, confirming the diagnosis.

The chorea was controlled with high doses (30 mg/day) of oral haloperidol. The depressive mood and psychotic symptoms remitted with Haloperidol and Venlafaxine. The Venlafaxine was suspended a few days later, because the patient developed moderate maniac symptoms. Later on, for reappearance of low mood, Sertraline was initiated with good response.

Cognitive functions were assessed using the Clock Drawing Test: she was able to draw the contour but could not draw numbers and clock hands. Mini-Mental State (MMS): 14/30 (the same result) and Montreal Cognitive Assessment (MOCA): 7/30.

At the moment of discharge to a special unit for rare diseases, she was euthymic, with proper conduct and without choreiform movements, although she still presented residual symptoms, namely, apathy, cognitive impairment, and overvalued ideas of ruin, conditioning severe limitations in her daily life, which made it impossible to live without continuous support. She will maintain follow-up by the Neuropsychiatry Team.

## 4. Discussion

We present a case of a 65-year-old woman that during the last six years was observed in different settings (mainly in the ES) by several professionals of diverse specialties. She presented with several symptoms, from depressive mood and anxiety to cognitive and behavior changes, as well as movement dysfunction. This variable clinical presentation led to multiple diagnostic hypotheses until HD was confirmed during her last hospitalization, during which she was evaluated by our Neuropsychiatric Team.

The first manifestation of the disease was depression, which is highly prevalent, occurring in up to 32–44% of HD patients, preceding the other symptoms from 2 to 20 years [11]. Depression is thought to be related to early degeneration of the medial caudate [11] and to degeneration in striatal circuits involving the frontal lobe and ventral anterior and medial dorsal nuclei of the thalamus [12]. In the case that we report, the depressive symptoms preceded the motor and cognitive dysfunction by about 3 years. Up to this point, a diagnosis of HD was highly improbable based only on depressive symptoms and without any family history. Moreover, the myocardial infarction at 63 years of age with the subsequent functional impairment confused the usual presentation of HD and added a stress factor that could justify the progressive worsening of depressed mood.

The early depressive symptoms were initially treated with Sertraline with some improvement. There is a lack of published evidence to guide pharmacological treatment of depression in HD patients, but some studies suggested a benefit with SSRIs and the SNRI Venlafaxine [12]. For that reason, during her second hospitalization, Venlafaxine was initiated with reversion of depressed mood. However, later on, our patient presented manic symptoms, interpreted as drug induced mania, and the antidepressive was discontinued. However, despite the fact that depressive episodes are much more frequent than manic episodes, HD is also considered a neurologic cause of mania [13] which could enhance the probability of secondary mania induced with Venlafaxine.

Her progressive noncompliance with medical advice and exams and multiple episodes of leaving the ERS against medical advice were probably early signs of behavioral and cognitive impairment. Dementia in HD is subcortical including deficits in processing speed, attention, visuospatial impairment, and dysarthric speech [12]. During the first hospitalization, these changes were interpreted as Pseudodementia in a 65-year-old woman, who had persistent depressed mood that had not responded to multiple antidepressants (probably due to therapeutic noncompliance) and mild cognitive impairment.

Movement dysfunction was evaluated at Neurology Consultation and AIU with an inconclusive clinical evaluation, and it was labelled as Conversion Disorder. The wide-based gait observed is a classical presentation of chorea in HD, but it is also similar to the one caused by cerebellar ataxia [14]. CT and Electromyography did not reveal any hypothesized lesion location that could explain the symptomatology. The choreiform movements can be worsened by stress factors, such as anxiety [15] which is another frequent psychiatric symptom of HD which she had complained of. The late-onset presentation (over 60 years) was another confounding factor for the diagnosis, due to the perceived low likelihood of HD at this age. Moreover, 94.4% of reported cases of LoHD had CAG repeat lengths of ≤44 (as our case) and, despite motor manifestations being the commonest initial presentation, 29.2% of the patients presented with nonmotor manifestations as the first clinical feature [15].

Regarding treatment, in this patient haloperidol was used successfully to address chorea, but there are several other approaches [12]. There is no available pharmacological treatment to stop the progression of HD, so treatment is focused on improving daily functioning [16]. Although there was a reduction in the severity of mood and movement dysfunction, our patient still manifests residual symptoms such as apathy. The latter is more frequent in advanced stages of the disease and is, among psychiatric symptoms, the most robustly associated with disease progression and cognitive and motor dysfunction [17].

## 5. Conclusions

Neuropsychiatric disorders often have overlapping and ambiguous presentations, constituting a diagnostic challenge. These could be more noticeable in individuals with LoHD, in which cognitive impairment, rather than chorea, may be the major source of disability.

Thereby, this case enhances the importance of effective collaboration between medical specialties that are dedicated to the study of the brain. The diagnosis of HD was finally achieved, only after the involvement of NT, showing that a multidisciplinary approach is a key factor in this kind of cases.

This case report also alerts clinicians for the need of a careful investigation of motor abnormalities in psychiatric patients, before attributing these symptoms to causes, such as iatrogenic effects or Conversion Disorder.

## References

[1] Pringsheim T., Wiltshire K., Day L., Dykeman J., Steeves T., Jette N. (2012). The incidence and prevalence of Huntington's disease: a systematic review and meta-analysis. *Movement Disorders*.

[2] Clark D., Danzl M. M., Ulanowski E. (2016). Development of a community-based exercise program for people diagnosed and at-risk for Huntingtons disease: A clinical report. *Physiotherapy Theory and Practice*.

[3] Garcia-Ruiz P. J., Garcia-Caldentey J., Feliz C., Del Val J., Herranz A., Martínez-Castrillo J. C. (2016). Late onset Huntington's disease with 29 CAG repeat expansion. *Journal of the Neurological Sciences*.

[4] Petit A.-C., Hozer F., Youssov K., Lavaud P., Hardy P., Mouaffak F. (2016). Differential response to ECT of psychotic and affective symptoms in Huntington’s disease: A case report. *The Journal of Neuropsychiatry and Clinical Neurosciences*.

[5] Paulsen J. S., Long J. D., Johnson H. J. (2014). Clinical and Biomarker Changes in Premanifest Huntington Disease Show Trial Feasibility: A Decade of the PREDICT-HD Study. *Frontiers in Aging Neuroscience*.

[6] Quaid K. A., Eberly S. W., Kayson-Rubin E., Oakes D., Shoulson I. (2017). Factors related to genetic testing in adults at risk for Huntington disease: the prospective Huntington at-risk observational study (PHAROS). *Clinical Genetics*.

[7] Wiatr K., Szlachcic W. J., Trzeciak M., Figlerowicz M., Figiel M. (2017). Huntington Disease as a Neurodevelopmental Disorder and Early Signs of the Disease in Stem Cells. *Molecular Neurobiology*.

[8] Paulsen J. S., Nehl C., Hoth K. F. (2005). Depression and stages of Huntington's disease. *The Journal of Neuropsychiatry and Clinical Neurosciences*.

[9] Gargiulo M., Lejeune S., Tanguy M.-L. (2009). Long-term outcome of presymptomatic testing in Huntington disease. *European Journal of Human Genetics*.

[10] van Duijn E., Craufurd D., Hubers A. A. M. (2014). Neuropsychiatric symptoms in a European Huntington's disease cohort (REGISTRY). *Journal of Neurology, Neurosurgery & Psychiatry*.

[11] Xu C., Yogaratnam J., Tan N., Sim K. (2016). Psychosis, treatment emergent extrapyramidal events, and subsequent onset of huntington's disease: A case report and review of the literature. *Clinical Psychopharmacology and Neuroscience*.

[12] Wyant K. J., Ridder A. J., Dayalu P. (2017). Huntington’s Disease—Update on Treatments. *Current Neurology and Neuroscience Reports*.

[13] Mendez M. F. (2000). Mania in neurologic disorders. *Current Psychiatry Reports*.

[14] Delval A., Krystkowiak P. (2010). Locomotion disturbances in Huntington's disease. *Revue Neurologique*.

[15] Chaganti S. S., McCusker E. A., Loy C. T. (2017). What do we know about late onset Huntington's disease?. *Journal of Huntington’s Disease*.

[16] Coppen E. M., Roos R. A. C. (2017). Current Pharmacological Approaches to Reduce Chorea in Huntington’s Disease. *Drugs*.

[17] Gregory S., Scahill R. I., Seunarine K. K. (2015). Neuropsychiatry and White Matter Microstructure in Huntington’s Disease. *Journal of Huntington’s Disease*.

